# District flood vulnerability assessment using analytic hierarchy process (AHP) with historical flood events in Bhutan

**DOI:** 10.1371/journal.pone.0270467

**Published:** 2022-06-24

**Authors:** Karma Tempa

**Affiliations:** Civil Engineering Department, College of Science and Technology, Royal University of Bhutan, Phuentsholing, Bhutan; Bristol University/Remote Sensing Solutions Inc., UNITED STATES

## Abstract

Flood hazards are common in Bhutan as a result of torrential rainfall. Historical flooding events also point to flooding during the main monsoon season of the year, which has had a huge impact in many parts of the country. To account for climate change patterns in flood hazards in Bhutan, 116 historical flood events between 1968 and 2020 for 20 districts were retrieved and reviewed. The preliminary review revealed that the frequency of flood occurrence has increased by three times in recent years. In this study, seven flood vulnerability (FV) indicators were considered. Five are the attributes of historical floods, classified into a number of incidents for flood events, fatalities, affected population, and infrastructure damages including economic losses. Additionally, the highest annual rainfall and existence of a flood map were other two indicators considered. Using historical data, flood hazard and impact zonation were performed. The analytic hierarchy process (AHP) method was employed to derive a multi-criteria decision model. This resulted in priority ranking of the seven FV indicators, broadly classified into social, physical/economic, and environmental. Thereafter, an indicator-based weighted method was used to develop the district flood vulnerability index (DFVI) map of Bhutan. The DFVI map should help researchers understand the flood vulnerability scenarios in Bhutan and use these to mediate flood hazard and risk management. According to the study, FVI is very high in Chhukha, Punakha, Sarpang, and Trashigang districts, and the index ranges between 0.75 to 1.0.

## Introduction

Climate change’s extreme events have triggered various natural hazards in Bhutan, causing huge impacts every year. Floods among other natural hazards continue to impend due to the effect of climate change [1]. Globally, flood hazards are one of the most destructive and costliest natural disasters [2–5]. Although flood damage is widespread and losses are enormous, due attention has yet to be paid in terms of hazard, vulnerability, and risk in Bhutan. The need for such a study has become important due to the limited number of studies conducted. Harm to property, deterioration of the environment, damage to infrastructure, and loss of life are some of the consequences of flood hazards [6]. Also, the impacts of floods are increasing as the result of cascading climate change scenarios, with predicted global flood exposure to escalate by three times by 2050 [7]. The combination of torrential rainfall and topography, land use and land-cover pattern, and demographic status further substantiates Bhutan to be highly vulnerable to flood hazards.

The potential impacts of flooding to exposed elements such as human beings, communities, properties, and the environment are assessed through flood vulnerability. According to the World Bank [8], a vulnerability assessment is conducted to understand how systems such as infrastructures could be damaged or destroyed, how service and business-chain supply could be interrupted and how the community could suffer through property loss, negative health impacts, and fatalities. Vulnerability has been well defined by Devkota [9] and according to this, a system’s predisposition to suffer damage during an extreme event is a representation of vulnerability and is therefore considered to be the magnitude of the impact on exposed system.

Based on local observations and drawing lessons from other countries, for Bhutan it is important to collate and analyse all the information linked to adverse effects of extreme weather phenomena over the past few decades that have led to many natural disasters, especially floods. The conceptual framework of flood vulnerability can be stated under various settings based on the purpose and perception of researchers [7]. In this study, the historical event-based framework was formulated to ascertain the hazard and impact levels in order to deduce and quantify vulnerability at the district level in terms of district flood vulnerability index (DFVI). The studies conducted by Diakakis [10] employed historical event-based hydrological data for flood history analysis and its contribution to hazard, while Burger et al. [11] accounted for climatic data. Discharge of past flood events was used by Sudhaus et al. [12], and the flow model for different return periods by Baky et al. [13], however, in some cases, historical events were often used only to enhance the knowledge or reconstruct the hydrological extremes [14–16]. In particular, historical traits are rarely mapped to assess the vulnerability of a region due to the absence of proper records. To this end, the current framework accounts for vulnerability driven by attributes of historical flood events that correlate with social, physical, economic, and environmental factors. The current study considered these factors as key indicators to derive flood vulnerability. An indicator-based approach has been widely used by many researchers and has been successfully employed in many regions at different scales, e.g., flood vulnerability indices at varying spatial scales [17], reducing the complexity of the flood vulnerability index [18], using the flood vulnerability index as a knowledge base for flood risk in urban areas [19], using the vulnerability index for urban flooding [20] and creating a new flood vulnerability index adapted for the pre-Saharan region [21]. Some also used a combination of flood vulnerability indicators and climate change impacts [22, 23]. Additionally, the literature review indicates a similar framework used at the provincial level in China using multidimensional flood vulnerability. The framework is based on data envelopment analysis, which considered indicators such as population, fatalities, agriculture, and economic vulnerability [24]. The current study presents the historical flood-event records between 1968 and 2020 that include flash floods of both river floods and surface water floods, and glacial lake outburst floods (GLOF). The historical sequence of flood events not only provides a comprehensive listing, but also highlights impacts and signals the prevalence from likely probable flood hazard scenario to heavy rain disasters [25]. By evaluating historical flood records, the frequency of flood-hazard events and subsequent impacts were mapped and vulnerability assessments were conducted to develop flood vulnerability indices at the district level using AHP and an open source QGIS software. According to Pathak et al. [7], the quantification of vulnerability is meant to build a more resilient community and help policy-makers facilitate prospective directions to frame out interventions effectively, serve as a pivotal tool for risk management and identify suitable climate change adaptation strategies. The research also attempts to address several UN sustainable development goals (SDGs), e.g., good health and well-being, building resilient infrastructure, having sustainable cities and communities, and combatting climate change [26].

Most of the districts in Bhutan are regularly hit by floods that cause tremendous loss of property and infrastructures, including loss of life. The mapping of flood vulnerability is important for handling flood problems. The main objective of this paper is to prepare a DFVI map for Bhutan. The analytic hierarchy process (AHP) has been employed to derive a multi-criteria decision model to determine the significance and influence of flood vulnerability indicators. The DFVI will provide insight into flood vulnerability and further attention can be paid to conduct in-depth study for the districts which are highly vulnerable to flood hazards. Additionally, a DFVI map will assist planners in adopting strategies at the district level for resource allocation, mitigation measures and enhancing resilience to flood hazards.

## Study area

**Fig 1:**
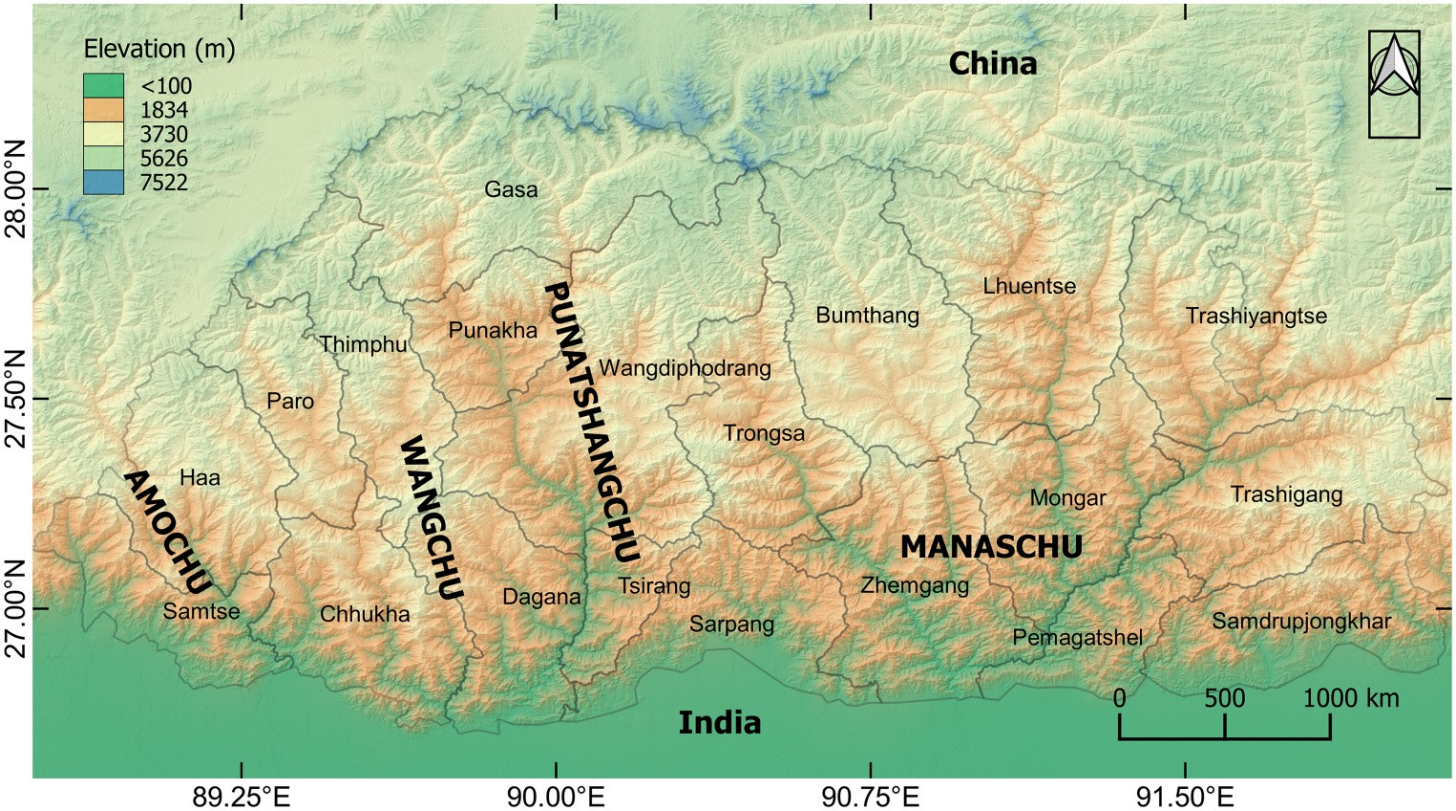
District level map of Bhutan showing main river basins.

**Fig 2:**
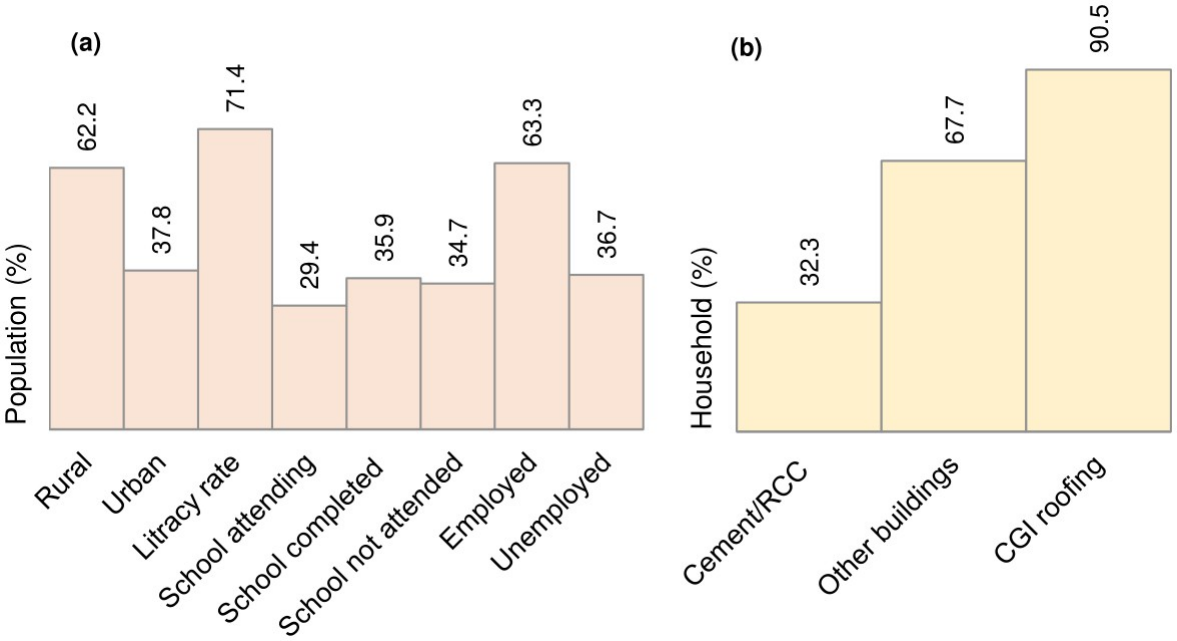
Segmentation of flood vulnerability attributes. (a) Demographic projection, (b) Household types.

Bhutan is situated in the Himalayan region, which extends between 26°45’N and 28°10’N latitudes and 88°45’E and 92°10’E longitudes, with three major zones of the southern foothills, inner Himalayas and higher Himalayas. The altitude ranges from 1000 m to more than 6000 m from sea level, as shown in Fig 1. According to the Population and Housing Census 2017, Bhutan’s population was projected to be 735,553 under 20 administrative districts and 205 local administrative units with 163,001 households [27]. The study area extends up to 38,394 km^2^ with a population density of 19.5 as of 2020 [28]. The study area is characterised by four major river basins, the Punatshangchu, Wangchu, Manaschu, and Amochu basins. According to the historical events, most of the regions at sub-catchment zones are affected by floods except for the Punatshangchu basin, which was hit by GLOF three times affecting downstream corridors of the basin such as Punakha, Wangdiphodrang, and Sunkosh (Tsirang). The impact was recorded highest in Punakha. The vulnerability exists mainly due to inhabitants that populate many pocketed rural areas in most of the sub-catchment zones and have a relatively low coping capacity compared to the urban area. Further, this population’s vulnerability is grossly upscaled with the additional infrastructure and service requirements, which are sheer attributes that come from livelihood needs. Fig 2 represents the demographic status of Bhutan that accounts for social and economic indicators. In the context of the proposed research, the demographic scenarios show substantial relative exposure to flood hazard risk (and other natural hazards). For instance, the rural population accounts for 62.2% of the total population, while other building categories such as masonry, mud and timber structures, and temporary houses, are as high as 67.7%. Also, 34.7% of the population has not attended schooling, and 36.7% is economically inactive apart from significant contributions through other attributes. These socio-economic attributes highlight Bhutan to be highly vulnerable to flood hazards, and susceptible to impending extremes of climate change.

## Materials and methods

### Historical flood data

The flood data were collected primarily through the National Centre for Hydrology and Meteorology (NCHM), the district administration office, and the archive of the national newspaper, *Kuensel*. Some of the data were also retrieved from respective web portals. From the data collection process, 116 historical annual flood events that occurred between 1968 and 2020 were retrieved (Table 1). The data retrieved were de-clustered as per the administrative boundaries (districts) and classified into the number of incidents for flood events, fatalities, affected population, and infrastructure damages. The economic loss was classified based on the extent of damages. Since the historical records do not have complete details of loss assessment, the cumulative extent of the damage level for the respective district was considered to classify the loss (very low to very high).

**Table 1:** De-clustered historical annual flood events since 1968 in 20 districts of Bhutan.

District	Year of event	# Events	Reported impacts ^a^			
			F	P	I_d_	L
Bumthang	2016	1	0	8,756	5	Very low
Chhukha	1990, 1991, 1996, 2000, 2005, 2008, 2009, 2010, 2015, 2016, 2019	16	41	27,545	71	Very high
Dagana	2009, 2015	2	0	350	4	Low
Gasa	2009, 2012, 2015, 2017	4	0	1,200	11	Low
Haa	1991, 2008	4	0	1,400	10	Very low
Lhuentse	1995, 2017, 2018, 2019	9	2	10,657	35	High
Mongar	2000, 2017	3	2	3,886	15	Low
Paro	1968	1	10	6,070	3	Low
Pemagatshel	2012	2	0	0	0	Very low
Punakha	1968, 1987, 1994, 2013, 2016	5	50	6,842	45	Very high
Samdrupjongkhar	1992, 2009, 2019	6	7	957	5	Low
Samtse	1993, 2000, 2008, 2016,	10	6	10,731	13	High
Sarpang	1968, 1996, 1999, 2010, 2012, 2015, 2016, 2017, 2019, 2020	15	28	20,911	103	Very high
Thimphu	1968, 2009, 2010, 2017	5	10	5,700	82	Moderate
Trashigang	1982, 1991, 1993, 1996, 2000, 2004, 2013, 2017, 2018	12	19	30,000	194	Very high
Trongsa	2020	2	0	0	0	Very low
Tsirang	1968	2	16	0	0	Very low
Wangdiphodrang	1968, 2015, 2016, 2020	4	0	3,500	12	Moderate
Trashiyangtse	1999, 2003, 2005, 2007, 2014	10	5	6,851	16	High
Zhemgang	N/A	3	0	1,860	25	Moderate

^a^ **F** = Fatalities, **P** = Population, **I**_**d**_ = Infrastructure damages; **L** = Economic loss

**Fig 3:**
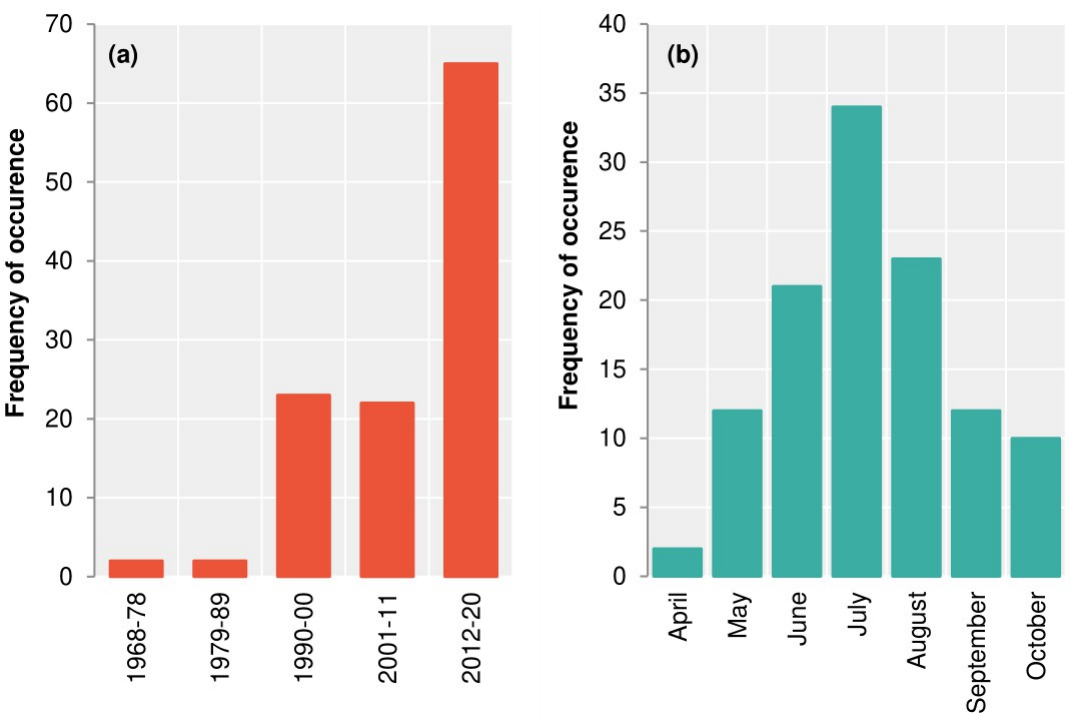
Frequency of flood occurrence. (a) Flood events in 10-year intervals, (b) Monthly flood events.

Preliminary assessment of the historical flood inventory indicates that most of the districts have recorded flood hazards of varying levels of severity and frequency of occurrence. Cataloguing flood events in 10-year intervals in Fig 3a, the highest number of flood events was recorded in the current decade with 65 flood histories. With the increasing trend, Bhutan has been suffering adverse manifestations of climate change-dependent, extreme weather conditions that have caused numerous floods, including GLOF. For example, the 1968, 1987, and 1994 Punakha floods were the major GLOF events within the Punatshangchu basin. Apart from GLOF, the majority of the other flood events account for the 113 floods to date across the country. Fig 3b indicates the highest number of flood events in July with 34 flood events.

### Rainfall data

**Fig 4:**
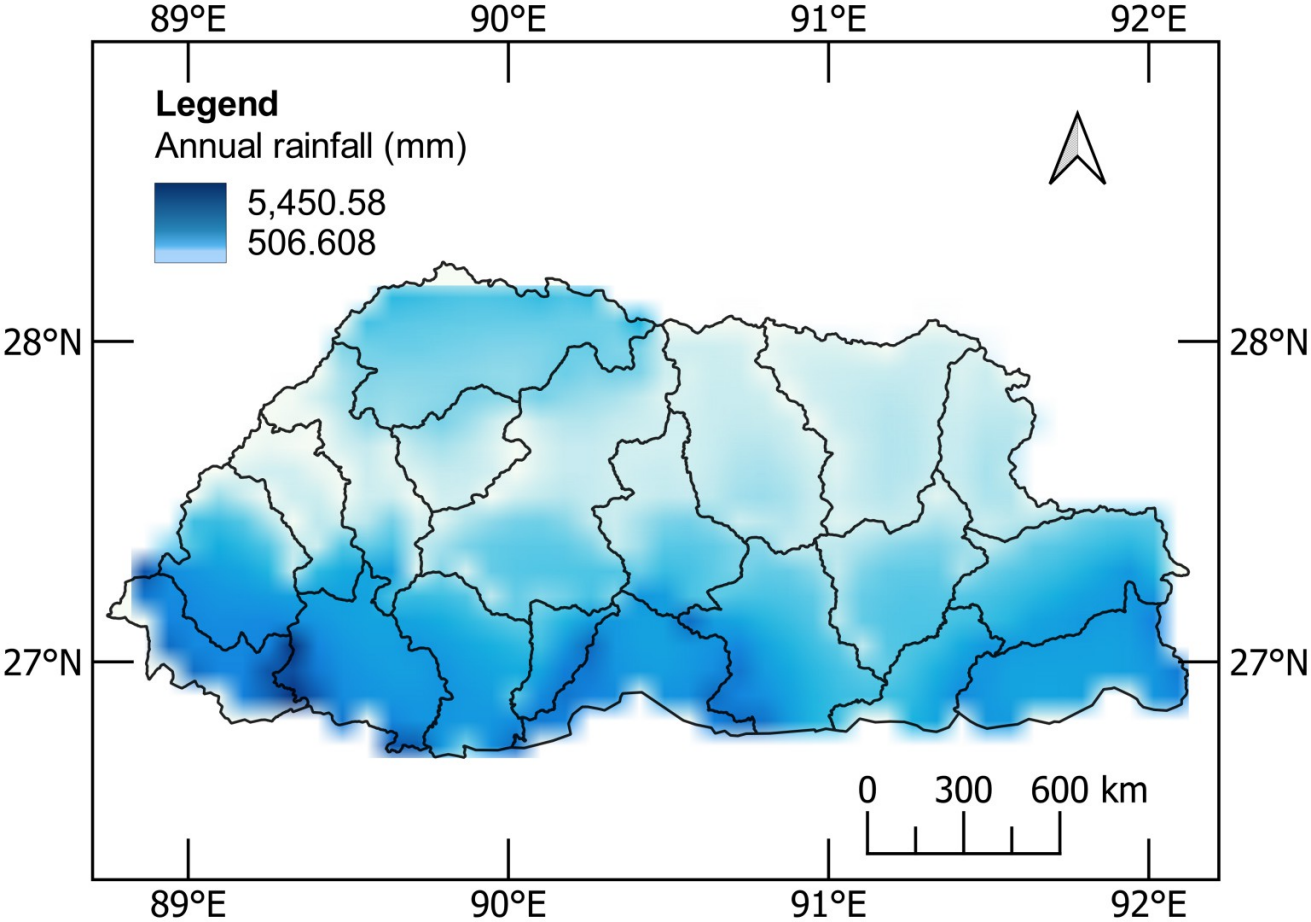
Spatial distribution of annual accumulated rainfall in 2020 against cumulative average rainfall between 1996–2019.

The precipitation in Bhutan manifests in monsoon characteristics and reveals significant variation, with the lowest total rainfall in January and the highest in July for most parts of the country [29]. According to Bhutan’s climatic record from 1996, the precipitation trend has been increasingly pronounced over the years, resulting in numerous extreme events [30–33]. From Bhutan State of the Climate 2020 [33], the highest rainfall records were used for all the districts, which served as an input parameter in QGIS to develop the rainfall map. The inverse distance weighting (IDW) method was employed for interpolating at random locations of the study area to show the spatial variations in five classes (Fig 4). The annual accumulated rainfall of 2020 is observed to be higher than the cumulative average annual rainfall between 1996 and 2019.

The southern belt of the country, which includes Sarpang, Samtse, Chhukha, and Samdrupjongkhar, usually received much higher rainfall, with moderate rainfall towards eastern and northern parts. The western and the central regions such as Bumthang, Paro, Punakha, Trongsa, and Wangdiphodrang experienced comparatively less rainfall. The annual cumulative rainfall is as high as 7220.30 mm in Sarpang and as low as 472.70 mm in Paro. In the extreme north of the greater Himalayan, Gasa has been also receiving moderately high rainfall annually. In 2020, Gasa recorded annual rainfall of 2508.30 mm.

### Development of the flood vulnerability index

To articulate FVI, an indicator-based weighted method was employed, which is widely used for flood system vulnerability assessment in many regions across the globe, e.g., a assessment of urban vulnerability towards floods using the indicator-based approach in Santiago de Chile [34], flood impact in the Mekong Delta, Vietnam [35], an indexing approach to assess flood vulnerability in coastal cities of Mazandaran, Iran [36], assessment of coastal sites in the UK for flood vulnerability with a combined physical and economic index [37], social vulnerability assessment for flood risk analysis [38], and a physical vulnerability assessment on flash floods using an indicator-based methodology [39] and followed by application of multi-criteria decision analysis (MCDA), with the AHP model adopted by many researchers to integrate the priority of indicators, e.g., [40–43]. DFVI in the present study was conceptually derived from Nasiri et al. [44]. Alternatively, geospatial analysis and remote-sensing (RS) techniques are widely used for flood vulnerability assessments, considering different indicators under respective regional settings. Such studies are possible due to the availability of geographical information system (GIS) platforms and RS products, e.g., a remote sensing and GIS-based flood vulnerability assessment in Jiangxi province, China [45], urban flood vulnerability using AHP and GIS [46], social vulnerability assessment of flood risk using GIS-based MCDA [47, 48], assessment of urban vulnerability to pluvial flooding using GIS applications [48], flood risk mapping using GIS and multi-criteria analysis [49], a GIS-based flood vulnerability assessment in Pasir Mas, Kelantan [50], a vulnerability assessment for flash floods using GIS spatial modelling in El-Arish city, North Sinai, Egypt [51], a flood vulnerability assessment using a GIS-based multi-criteria approach in Attica region [52], and a coastal flooding risk assessment using a GIS-based spatial MCDA approach [53]. The outline of the method employed in the current study can be divided into four parts as follows:

1. Identify main goal (weighting or score assignment to FV indicators according to the level of hazard and impact)
2. Formulate criteria (social, physical/economic, and environmental)
3. Priorities sub-criteria by AHP pair-wise comparison (population, fatalities, existence of flood map, loss and infrastructure damages, rainfall, and flood events)
4. Aggregate weighting of criteria to vulnerability scale (very high, high, medium, low, and very low)

The historical data provided us with the attributes of flood hazards and impacts, which were broadly categorised under the three domains of social, physical/economic, and environmental factors, which served as the main criteria. Corresponding sub-criteria were identified and classified into five classes. These account for exposure and susceptibility to flood vulnerability. The existence of a flood map specifies the resilience indicator for a particular district. The FV indicators are as shown in Table 2.

**Table 2:** FV indicators adopted for the study.

Indicators	Abbreviation	Unit	Criteria	FV factor
Social	F	Number	Fatalities	Susceptibility
	P	Number	Population	Exposure
	F_m_	Yes/No	Existence of flood map	Resilience
Physical/Economic	L	Class	Loss	Exposure
	I_d_	Number	Infrastructure damages	Exposure
Environmental	A_R_	mm	Annual rainfall	Susceptibility
	F_e_	Number	Frequency of events	Susceptibility

**Fig 5:**
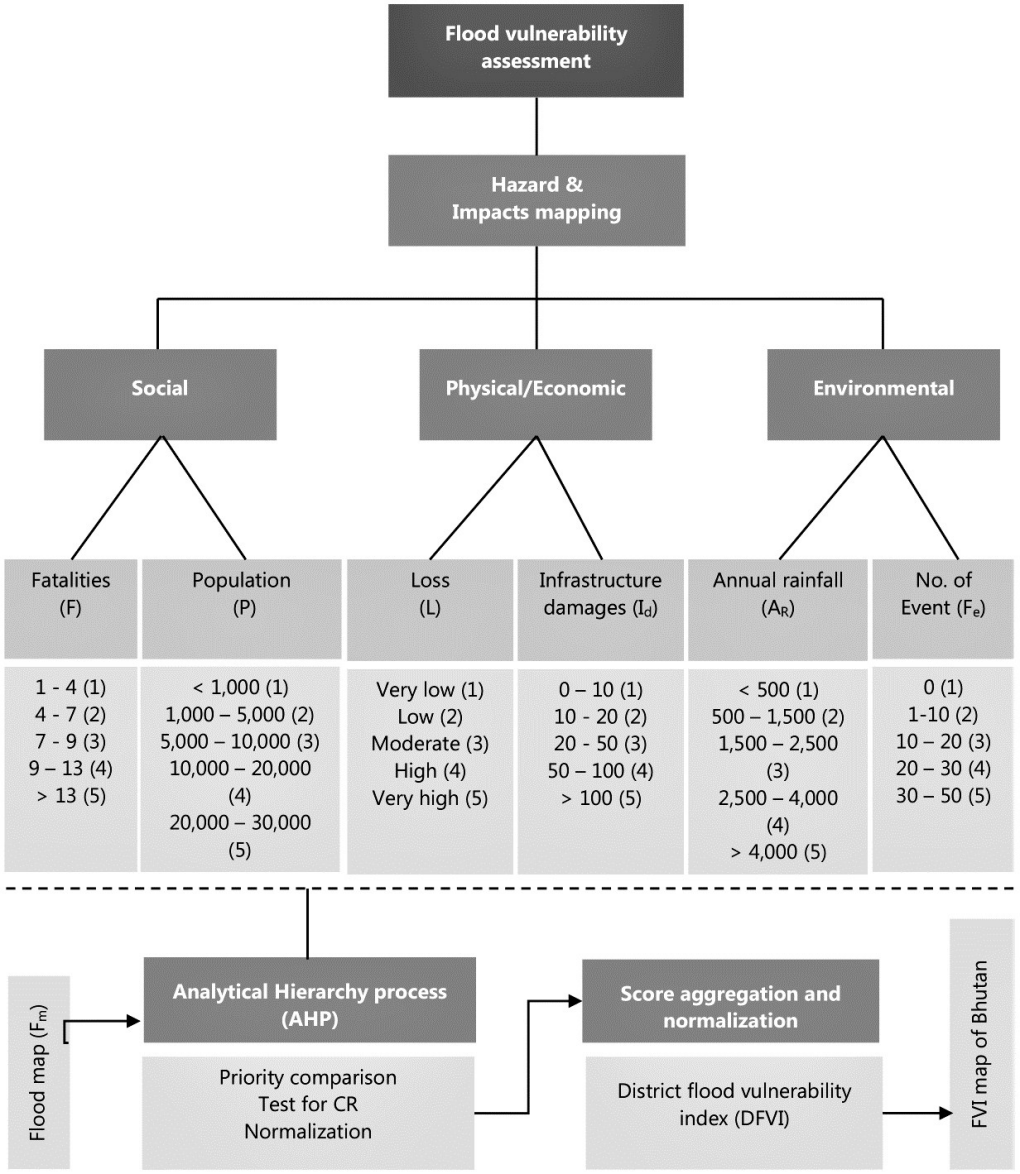
Methodological flowchart based on historical event-based indicators and application of AHP.

After de-clustering the historical flood data records, the score was assigned to the corresponding class on an ascending order of scale from 1–5. Such a scale of the severity level of hazard or impacts was also used by Blistanova et al. [54], and many others elsewhere. Initially, this scale system was defined by Mchaughlin and Cooper [55], with ‘5’ contributing most strongly to vulnerability, and ‘1’ contributing least. The results of classification are subsequently utilised in developing hazard and impact maps at the district level of Bhutan. FV indicators were used in AHP to define pair-wise comparison to obtain priority indices which were then applied to computed DFVI. The study consists of seven FV indicators used in the AHP model under the umbrella of three base criteria. Details of criteria, sub-criteria, weighting, and the methodological process are presented in Fig 5.

A multi-objective AHP developed by Saaty [56] employs multi-criteria decision analysis that uses a pair-wise comparison process to efficiently evaluate complex decisions by constructing the judgement matrix with the scale of absolute numbers 1–9 (Table 3). AHP uses hierarchical structures to represent a problem and then develops priorities for alternatives based on the judgement of the user [57]. The AHP model involves constructing a pair-wise matrix, eigenvalue and weighting coefficient calculation, and allows a check for consistency of the priority ranking by calculation of consistency ratio (CR) [58]. The consistency indices (CI) and CR of a given choice are calculated using Eqs 1 and 2.

$$\text{CI}=\left(\frac{\lambda_{\text{max}}-\text{n}}{\text{n}-1}\right)$$ (1)

$$\text{CR}=\text{CI}\left(\frac{1}{\text{RI}}\right),$$ (2)

where λ_max_ is the maximum eigenvalue of the pair-wise comparison vector and *n* is the number of attributes. The random index (RI) is as indicated in Table 4.

**Table 3:** The fundamental scale of various compared elements.

Scale	Judgement of preference	Description
1	Equally important	Two factors contribute equally to the objective
3	Moderately important	Experience and judgement slightly favour one over the other
5	Important	Experience and judgement favour one over the other
7	Very strongly important	Experience and judgement strongly favour one over the other
9	Extremely important	The evidence favouring one over the other is of the highest possible validity
2, 4, 6, 8	Intermediate preference between adjacent scales	When compromise is needed

**Table 4:** Random consistency index [57].

n	1	2	3	4	5	6	7	8	9	10
RI	0	0	0.58	0.9	1.12	1.24	1.32	1.41	1.45	1.49

## Results and discussion

### Flood hazard zonation

**Fig 6:**
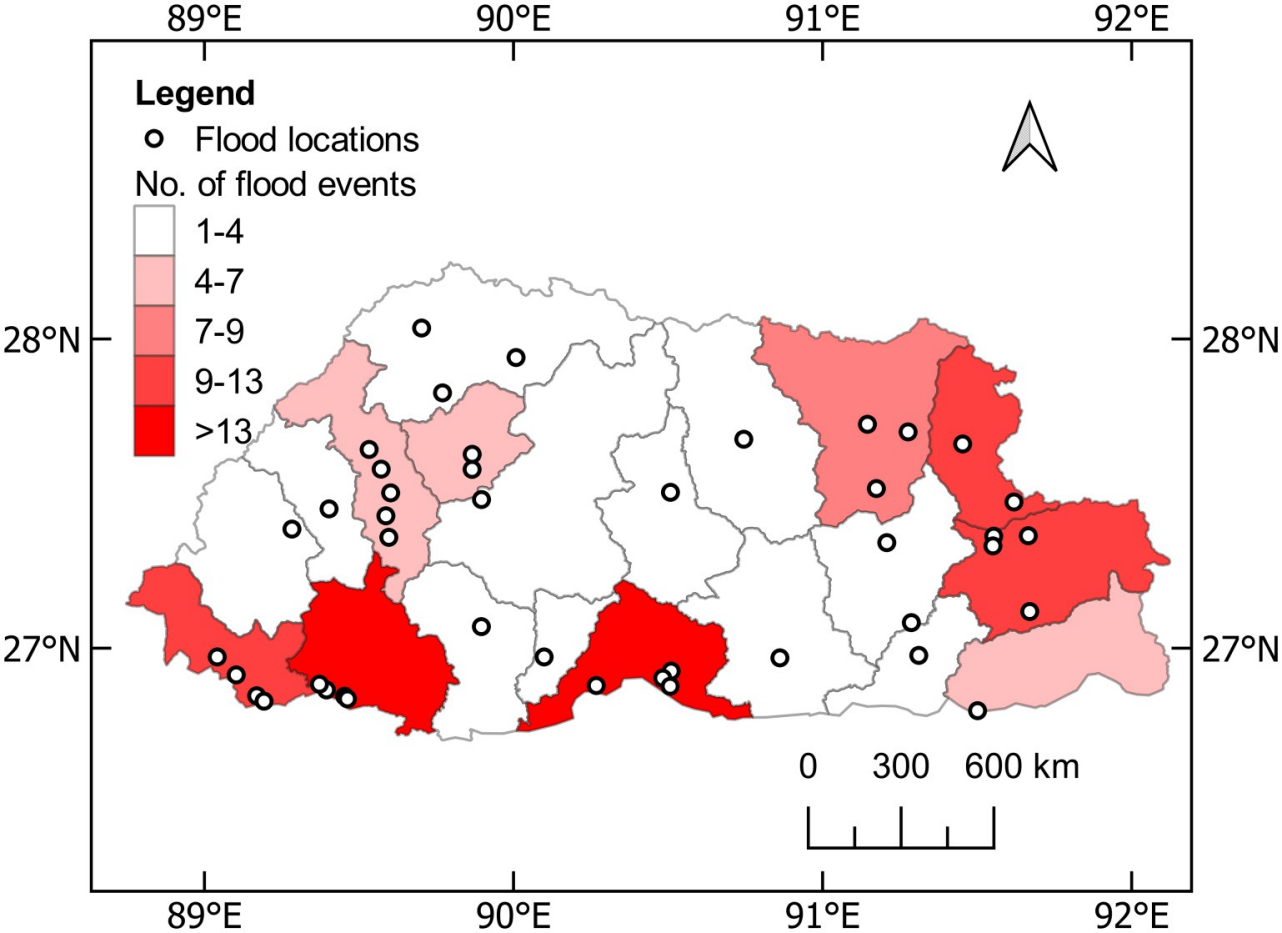
District-level flood hazard zonation of Bhutan and locations of the major flood events.

The record of major events accounts for 116 historical floods in Bhutan between 1968 and 2020. The district-wide distribution of historical flood hazards is shown in Fig 6. The map also depicts the locations of the major flood events including recurrent events. Since 1990, the Chhukha district has been hit by the highest number of 16 flood events. A major flood disaster occurred in 1996 at Pasakha, when the Barsachu river flooded. The flood damaged more than 25 residential buildings, incurring losses of more than Nu. 30 million. Similarly, Phuentsholing city under the same district was also devasted by the 2000 Dhuti Khola flood and the 2016 Amochu flood.

The frequency of floods in southern regions of the country shows a similar trend. Sarpang and Samtse experienced 10 and 15 major flood events respectively. The eastern part of the country also recorded major flood events, with 9 in Lhuentse, 10 in Trashiyangtse, and 12 in Trashigang. Other districts observed moderately few flood events, except for the Punakha district, which was hit by three major GLOF events. According to a study conducted by Gurung et al. [59], Punakha remains highly susceptible to GLOF due to active Lemthang Tsho and its association with climate change.

### Flood hazard impacts

**Fig 7:**
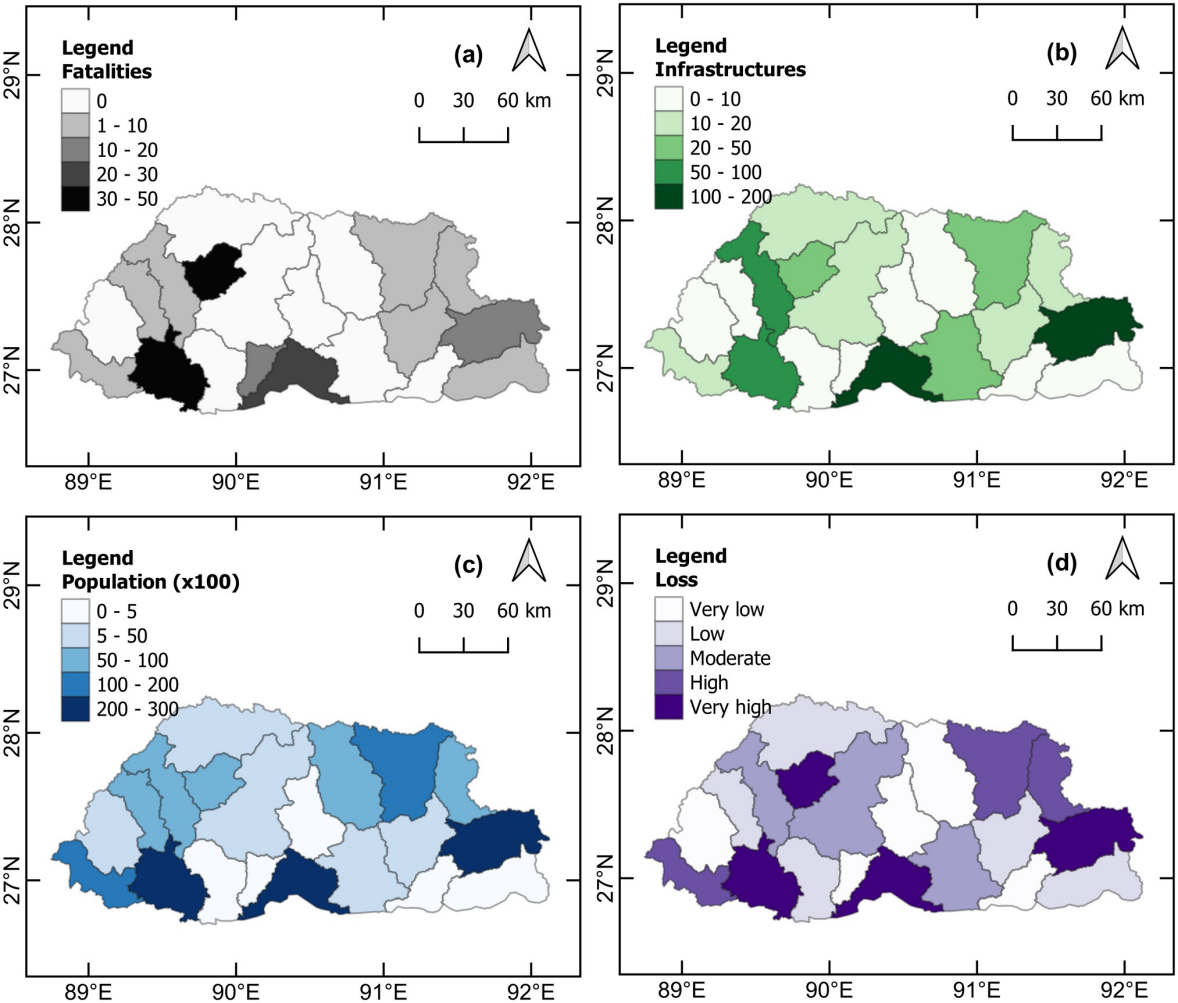
District-level hazard impacts due to historical flood events in Bhutan. (a) Fatalities, (b) Infrastructure damage, (c) affected population, (d) Physical/economic loss.

An assessment of four flood impacts (fatalities, infrastructure damages, affected population, and loss) was undertaken based on historical flood events (Fig 7). Such an impact assessment not only gives a detailed insight into flood characteristics but is also helpful in providing information on warning and evacuation [60, 61]. To assess the flood hazard impact levels, the study considered cumulative impacts for all the FV indicators.

Fig 7a shows the number of fatalities within each district due to flooding. The Chhukha, Punakha, and Sarpang districts recorded a greater number of fatalities, followed by five districts in the east. It should be noted that the greater number of fatalities is a consequence of a higher number of flood events, including major floods due to GLOF in Punakha. The central and extreme northern regions recorded a comparatively lesser number of flood events and fatalities. The Paro and Thimphu districts in the west recorded moderately higher fatalities.

Infrastructure damage includes the total number of damage incidences of residential, commercial, industrial, and monumental buildings, retaining structures, water supply systems and tanks, instrumentation stations, bridges, roads, and irrigation channel segments. As shown in Fig 7b, the infrastructure damages were highest in the Sarpang and Trashigang districts, followed by a significant number in 10 districts. Eight other districts recorded a minimal number of infrastructure damages between 1 and 10.

Bhutan’s population is densely populated, especially in urban areas (main district) under various sub-catchments. The total affected population was assessed based on the location of flood events in each district and the results are shown in Fig 7c. The assessment results indicate a lesser number of populations being affected in fewer districts. This shows that the affected populations are dependent not only on the frequency of flood occurrence but on the collateral impacts. For instance, the damage or collapse of a bridge could result in road network disruption, and the population in nearby local administrative units would thus be affected.

In the absence of detailed information, qualitative judgement was employed to classify economic losses (Fig 7d). The records of such information broadly describes damages and does not have complete assessment records. For example, loss of household properties or belongings, damages to agricultural farmland (wetland), dry land, cash crops, cattle, fruit trees, industrial setup, etc. These losses are often difficult to consider for an accurate valuation in terms of monetary value and that the damages have occurred in different time frames. Usually, a field assessment is a must to accurately estimate the actual loss. Hence, we rated the loss based on the overall damage extent from very low to very high in five classes. However, since an indicator-based weighted method was applied, the class and score approximately accede to these economic losses. The actual damage loss model accounts for the combination of direct tangible and indirect tangible costs including intangible costs, where effects are felt by the society, but the accompanying losses and damages are difficult to value in terms of monetary cost [62], something that is also highlighted and discussed in [63, 64]. Here, economic losses are considered similar to intangible costs.

### Flood vulnerability index

The indicator-based weighted method employs expert judgement to form pair-wise comparisons in AHP model execution. In the current study, pairwise priority was employed based on both expert judgement and the score of the class, derived from records of historical events. Table 5 represents the FV indicator scores for the Chhukha district that are used in the AHP pair-comparison ranking matrix, with the highest weighting of 5 in four criteria and a weighting of 4 for infrastructure damage. This also indicates that the FV indicators in the Chhukha district are one of the highest contributing attributes of historical flood events.

**Table 5:** Class and scores for FV attributes for Chhukha district.

Social		Physical/economic		Environmental		Score
F	P	L	I_d_	A_R_	F_e_	
-	-	-	-	-	-	1
-	-	-	-	-	-	2
-	-	-	-	-	-	3
-	-	-	50 to 100	-	-	4
30 to 50	20,000 to 30,000	Very high	-	> 4,000	> 13	5

These scores are correlated to pair-wise comparison for the AHP model to assign significance of importance with a scale of 1, 3, 5, 7, and 9 respectively, while a scale of 2, 4, 6, and 8 indicates values of intermediate importance. Conversely, inverse comparisons of less important variables were assigned 1 to 1/9. The priority matrix for the Chhukha district is shown in Table 6 to illustrate the importance of each FV indicator to overall flood vulnerability. The pair comparison matrix was formulated with 21 comparisons and a similar procedure was applied in 19 other districts. As a result of the AHP method, the district-level priority ranking for seven FV indicators is presented in Table 7. Each district displayed a unique correlation among the FV indicators. The economic loss, affected population and annual rainfall dominates the flood vulnerability scenarios, followed by infrastructure damage, and fatalities on account of the frequency of flood events. Some flood events indicate destructive nature of floods against frequency of occurrence. For example, Haa, Paro, Punakha, Thimphu, Wangdiphodrang, and Zhemgang are some of the districts which experienced such flood events. On the contrary, some of the other districts were impacted due to a higher number of flood events and rainfall intensity, e.g., Chhukha, Sarpang, Samtse Trashigang, and Trashiyangtse.

**Table 6:** AHP priority matrix for DFVI indicators for Chhukha district.

AHP priorities	F	P	F_m_	L	I_d_	A_R_	F_e_
Fatalities	1.00	1.00	1/2	1.00	3.00	1.00	1/3
Population	1.00	1.00	3.00	1.00	1.00	1.00	1.00
Flood map	2.00	1/3	1.00	1/2	1/2	1/3	1/3
Loss	1.00	1.00	2.00	1.00	2.00	1.00	1.00
Infrastructure damage	1/3	1.00	2.00	1/2	1.00	0.33	1.00
Annual rainfall	1.00	1.00	3.00	1.00	3.00	1.00	1.00
Frequency of events	3.00	1.00	3.00	1.00	1.00	1.00	1.00
Σ	9.33	6.33	14.50	6.00	11.50	5.66	5.66

**Table 7:** District-level priority rankings of FV indicators (%) and corresponding CR values.

Basin/sub-basin	District	F	P	F_m_	L	I_d_	A_R_	F_e_	CR (%)
Wangchu	Haa	11.4	17.0	11.8	17.2	14.9	16.3	11.4	3.5
	Paro	14.9	19.5	6.9	25.1	11.2	11.2	11.2	7.1
	Thimphu	15.2	15.2	6.4	15.2	26.3	6.7	15.0	7.4
	Chhukha	13.5	15.3	8.6	15.5	10.4	18.0	18.7	9.8
Punatshangchu	Dagana	6.0	14.4	7.9	22.5	13.9	23.9	11.4	9.8
	Gasa	8.0	14.0	11.5	20.0	20.0	18.6	7.9	8.4
	Punakha	31.2	12.4	3.3	30.7	6.5	7.6	8.3	2.5
	Tsirang	20.7	8.7	4.7	8.5	7.4	42.0	8.0	2.3
	Wangdiphodrang	5.3	28.9	7.5	27.8	12.6	12.6	5.3	5.1
Chamkharchu	Bumthang	8.7	26.1	5.9	9.8	10.3	28.8	10.4	6.4
Mangdechu	Trongsa	7.4	7.7	7.7	21.0	8.8	20.5	26.9	5.2
	Zhemgang	5.1	11.8	6.6	29.3	20.4	20.6	6.2	8.4
Nyera Amari	Samdrupjongkhar	10.8	8.7	3.1	14.8	4.8	48.4	9.4	4.0
Jaldakha	Samtse	4.9	7.4	3.5	20.5	5.3	35.3	23.1	9.0
Aiechu	Sarpang	6.3	15.3	5.2	18.3	18.3	18.3	18.3	4.8
Drangmechu	Lhuentse	9.7	19.8	4.8	27.4	20.1	6.8	11.4	9.7
	Pemagatshel	16.0	12.3	5.8	31.9	16.8	10.2	7.0	1.6
	Mongar	17.2	16.1	7.7	17.2	17.2	17.2	7.4	2.4
	Trashigang	6.1	24.7	5.1	24.0	24.0	5.4	10.7	5.0
	Trashiyangtse	7.4	13.8	5.3	26.6	7.8	8.2	30.9	7.7

The priority ranking matrix requires a consistency ratio (CR) of less than 10%. In the few instances where the CR was more than 10%, pair-wise comparison indicators having the same score were re-prioritised based on the significance of the flood event and expert judgement. This re-prioritisation was observed to have a close correlation among the same group at the individual district level. The CR in AHP model analysis for 20 districts (Table 7) was achieved between 1.60% and 9.8%.

The representative normalised flood indicators for the Chhukha district are presented in Table 8. For existence of a flood map, 1 (Yes) was indicated, and 0 (No) was indicated for districts which do not have a flood map. The existence of a flood map is considered as a resilience indicator; however, the priority differed in each district based on significance of other indicators.

**Table 8:** Normalisation of vulnerability indices for Chhukha district.

Normalised	F	P	F_m_	L	I_d_	A_R_	F_e_
Fatalities	0.11	0.16	0.03	0.17	0.26	0.18	0.06
Population	0.11	0.16	0.21	0.17	0.09	0.18	0.18
Flood map	0.21	0.05	0.07	0.08	0.04	0.06	0.06
Loss	0.11	0.16	0.14	0.17	0.17	0.18	0.18
Infrastructure damage	0.04	0.16	0.14	0.08	0.09	0.06	0.18
Annual rainfall	0.11	0.16	0.21	0.17	0.26	0.18	0.18
Frequency of events	0.32	0.16	0.21	0.17	0.09	0.18	0.18
Σ	1.00	1.00	1.00	1.00	1.00	1.00	1.00

**Fig 8:**
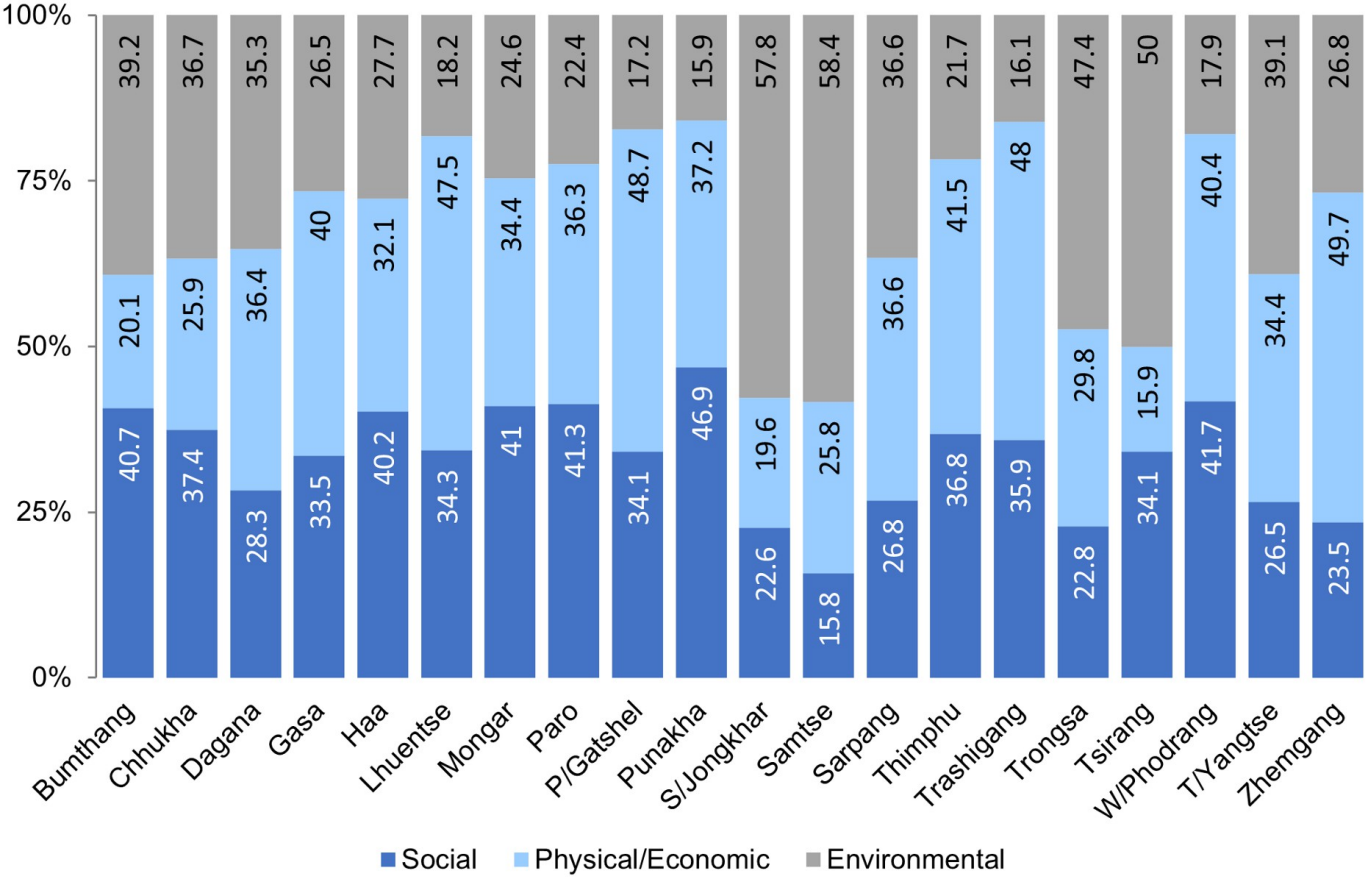
Priority ranking of base indicators under each district as an outcome of AHP.

The consolidated priority ranking of the AHP model for 20 districts in Fig 8 shows the unique correlation among indicators under each district, for example, in Samtse and Samdrupjongkhar districts, the environmental indicators of rainfall and frequency of flood account for ~58%, imposing high susceptibility, compared to social and physical exposure indicators, which hold ~16 to 25% respectively. On the contrary, in Sarpang and Chhukha, with a ~25 to 37% contribution by all indicators, both the susceptibility and exposure levels have relatively similar impacts and these districts remain highly vulnerable to flood hazards. Also, Punakha displays a high social vulnerability of 46.9% and the overall vulnerability remains at an all-time high, as it falls in the regions of the largest Punatshangchu river basin with the potential for GLOF outbreaks. The Samtse district also recorded a greater number of floods with environmental indicators showing up to 58.4%, with flood vulnerability as high. However, the social exposure and impact are comparatively lower. Bumthang, Pemagatshel, Paro, and Wangdiphodrang remain high to flood vulnerability in terms of social and physical exposure, however, the overall vulnerability remains at low due to lesser rainfall pattern and flood events. In such a case, the consequence of a major flood event could be devastating. The rest of the districts remain at an all-time low to flood hazard with few seasonal impacts.

The DFVI of each district was computed based on the general flood vulnerability index (FVI) formula (Eq 3), where, E, S, and R indicate exposure, susceptibility, and resilience respectively. This base formula is further re-generalised in Eq 4 to account for the number of indicators involved under each criterion, and DFVI is obtained by combining all the indicators using Eq 5 for each district. The results are presented in Table 9. Finally, the aggregated DFVI for each district is obtained by taking the sum of social, physical/economic, and environmental criteria using Eq 6 and normalising it by the maximum aggregated sum [11, 44] as supplied in Table 10.

$$\text{FVI}=\frac{\text{E}\;\text{x}\;\text{S}}{\text{R}},$$ (3)

$$\text{y}=\sum_{\text{i}=1}^{\text{n}}{\text{W}}_{\text{i}}{\text{X}}_{\text{i}},$$ (4)

$$\text{DFVI}=\frac{\sum_{\text{E}=1}^{\text{n}}{\text{X}}_{\text{E}}{\times \text{W}}_{\text{E}}\text{x}\sum_{\text{S}=1}^{\text{n}}{\text{X}}_{\text{s}}{\times \text{W}}_{\text{s}}}{\sum_{\text{R}=1}^{\text{n}}{\text{X}}_{\text{R}}{\times \text{W}}_{\text{R}}},$$ (5)

**Table 9:** Assigned normalised weights to DFVI indicators for 20 districts.

District/FVI	F	P	F_m_	L	I_d_	A_R_	F_e_
Bumthang	0.09	0.26	0.06	0.10	0.10	0.28	0.10
Chhukha	0.14	0.15	0.08	0.16	0.11	0.18	0.18
Dagana	0.06	0.14	0.08	0.23	0.14	0.24	0.12
Gasa	0.08	0.15	0.12	0.19	0.19	0.19	0.08
Haa	0.12	0.16	0.12	0.16	0.14	0.17	0.12
Lhuentse	0.10	0.25	0.05	0.26	0.14	0.08	0.12
Mongar	0.17	0.16	0.08	0.17	0.17	0.17	0.08
Paro	0.15	0.19	0.07	0.25	0.11	0.11	0.11
Pemagatshel	0.09	0.23	0.10	0.10	0.11	0.27	0.11
Punakha	0.31	0.12	0.03	0.30	0.07	0.08	0.09
Samdrupjongkhar	0.11	0.09	0.03	0.15	0.05	0.47	0.10
Samtse	0.05	0.08	0.04	0.20	0.06	0.34	0.23
Sarpang	0.06	0.16	0.05	0.18	0.18	0.18	0.18
Thimphu	0.15	0.15	0.06	0.15	0.26	0.07	0.15
Trashigang	0.06	0.25	0.05	0.24	0.24	0.06	0.11
Trongsa	0.08	0.08	0.08	0.19	0.09	0.22	0.25
Tsirang	0.21	0.09	0.05	0.09	0.07	0.42	0.08
Wangdiphodrang	0.05	0.29	0.08	0.27	0.13	0.13	0.05
Trashiyangtse	0.07	0.16	0.04	0.27	0.07	0.07	0.31
Zhemgang	0.05	0.12	0.07	0.29	0.21	0.20	0.06

**Table 10:** Final DFVI for all districts.

District/DFVI	Social	Physical /Economic	Environmental	Total, DFVI	Normalized, DFVI
Bumthang	0.75	0.01	0.06	0.82	0.11
Chhukha	6.41	0.33	0.83	7.56	1.00
Dagana	0.11	0.06	0.05	0.22	0.03
Gasa	0.20	0.14	0.05	0.39	0.05
Haa	0.16	0.02	0.04	0.22	0.03
Lhuentse	3.84	0.44	0.06	4.33	0.57
Mongar	1.45	0.06	0.03	1.53	0.20
Paro	2.52	0.14	0.01	2.67	0.35
Pemagatshel	0.21	0.16	0.12	0.49	0.06
Punakha	5.96	0.29	0.03	6.28	0.83
Samdrupjongkhar	0.63	0.02	0.45	1.10	0.14
Samtse	0.88	0.12	1.58	2.58	0.34
Sarpang	4.58	0.82	0.82	6.23	0.82
Thimphu	2.17	0.49	0.02	2.68	0.35
Trashigang	4.47	1.42	0.05	5.94	0.79
Trongsa	0.08	0.02	0.11	0.21	0.03
Tsirang	1.15	0.01	0.10	1.26	0.17
Wangdiphodrang	0.60	0.21	0.01	0.83	0.11
Trashiyangtse	1.78	0.16	0.18	2.12	0.28
Zhemgang	0.19	0.55	0.03	0.76	0.10

where W_i_ is the weighted score and X_i_ represents priority indices for corresponding indicators respectively;

$${\text{DFVI}}_{\text{Total}}={\text{DVFI}}_{\text{Social}}+{\text{DVFI}}_{\text{Physical}/\text{Economic}}+{\text{DVFI}}_{\text{Environmental}}.$$ (6)

**Fig 9:**
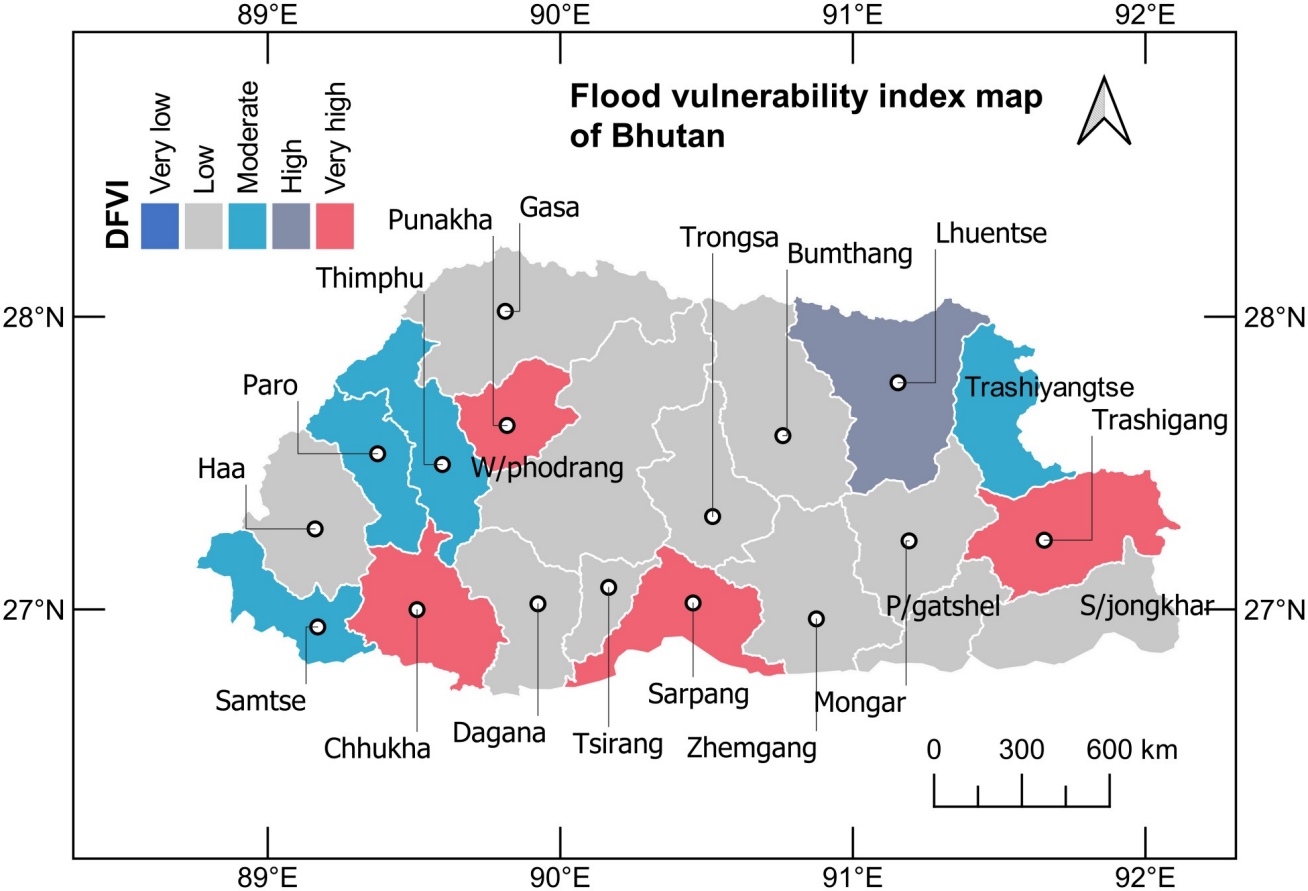
District flood vulnerability index (DVFI) map of Bhutan.

The final obtained DFVI is scaled to define the vulnerability level [21] as presented in Table 11. The scaled vulnerability index provides flood vulnerability designation for each of the districts ranging between 0–0.01 as very low vulnerability to flooding, and 0.75–1.0 as very high vulnerability to flooding, with other intermediate values. The study indicates four districts with very high flood vulnerability, including two in the southern region, and one each in the east and west. One of the districts falls in the category of high vulnerability, another four in the medium category, and 11 districts in the low-flood vulnerability category. Districts with higher DFVI in the east, west and southern region of Bhutan remain constantly vulnerable to floods compared to other districts that are primarily contributed by susceptibility to a high frequency of events and heavy rainfall. As a result of the study, the DFVI map of Bhutan is presented in Fig 9.

**Table 11:** Flood vulnerability ranking.

Index value	Vulnerability level	District	No (s).
< 0.01	Very low	Nil	0
0.01–0.25	Low	Bumthang, Gasa, Haa, Dagana, Mongar, Pemagatshel, Samdrupjongkhar, Trongsa, Tsirang, Wangdiphodrang, Zhemgang	11
0.25–0.50	Moderate	Paro, Samtse, Thimphu, Trashiyangtse	4
0.50–0.75	High	Lhuentse	1
0.75–1	Very high	Chhukha, Punakha, Sarpang, Trashigang	4

## Conclusions

In this paper, an assessment of historical flood events was undertaken and seven flood vulnerability indicators were identified under three broad categories of social, physical/economic, and environmental attributes. The multi-criteria decision analysis was carried out using AHP to formulate the influence of FV indicators to flood vulnerability in each district. The flood vulnerability index was computed based on an indicator-based weighted method to obtain the district flood vulnerability map of Bhutan. The following concluding remarks and recommendations are presented.

- According to the study, attributes of flood history show a unique function of susceptibility and exposure to flood vulnerability that play an important role in predicting the flood vulnerability index.
- Social indicators dominated the flood vulnerability in Chhukha, Punakha, Sarpang, and Trashigang, with 0.75–1.0 FVI, followed by Lhuentse with 0.57 FVI due to the high number of fatalities and the affected population. The Paro, Thimphu, and Trashiyangtse districts are also moderately high to social vulnerability to flood, with FVIs ranging between 0.25–0.50, while in the Samtse district, environmental indicators indicate a higher margin of flood vulnerability due to heavy rainfall and the significant number of flood events. The Bumthang, Dagana, Gasa, Haa, Mongar, Pemagatshel, Samdrupjongkhar, Trongsa, Tsirang, Wangdiphodrang, and Zhemgang districts remain at an all-time low to flood vulnerability. These districts receive comparatively low rainfall, with few flood histories. However, the study shows that exposure to flood vulnerability cannot be ruled out in these regions and FVI ranges between 0–0.1. The districts with a high vulnerability index indicate a very high possibility of reoccurrence of such flood events associated with extreme climatic conditions that are cascading every year.
- Social indicators significantly account for 15.8% to 46.9% of flood vulnerability. An in-depth study on social vulnerability of districts with high FVI is highly recommended to accurately assess the exposure and susceptibility level to flood hazards in Bhutan.
- The FVI developed in this research study can be used as a tool to strategise district-wise flood risk management and preparedness plans by the relevant stakeholders.

## Supporting information

S1 File (RAR)
